# Perceived Social Discrimination, Socioeconomic Status, and Alcohol Consumption among Chinese Adults: A Nationally Representative Study

**DOI:** 10.3390/ijerph17176043

**Published:** 2020-08-20

**Authors:** Jiafeng Gu, Xing Ming

**Affiliations:** 1 Institute of Social Survey Study, Peking University, Beijing 100871, China; 2 School of Sociology and Political Science, Shanghai University, Shanghai 200444, China; coclover@shu.edu.cn

**Keywords:** alcohol consumption, unfair treatment, perceived social discrimination, socioeconomic status, logistic model

## Abstract

Perceived social discrimination in China has significant effects on drinking behavior. This finding was reached through multivariate logistic regression analysis of a sample of 22,566 adults in the 2016 China Family Panel Studies (CFPS). This was a cross-sectional study conducted with computer-assisted face-to-face interviews to assess alcohol drinking problems and associated factors among Chinese adults. The proportion of adults prone to alcoholism tends to be higher in eastern than central China, and higher in central than western China. Furthermore, gender discrimination and delays in government interactions as a result of unfair treatment have a positive and significant effect on individuals’ drinking. The alcohol consumption rate among Chinese men is about 13 times that of Chinese women. Additionally, older people have a stronger tendency to drink alcohol. In terms of education, those with lower education levels are more prone to alcoholism than those with higher education levels. Regarding marital status, those who are married are more prone to alcoholism than those who are not. Further, those who have been diagnosed with a chronic disease within the past six months are less prone to alcoholism than those without such diagnosis. People with an annual income between 50,000 and 150,000 yuan are more prone to alcoholism than those with an income under 50,000 yuan. Groups that have experienced unequal treatment in public services are also more prone to alcoholism than those who do not suffer such unequal treatment.

## 1. Introduction

In today’s world, alcohol poses an immense threat to physical and mental health. In 2016, alcohol was the seventh leading cause of death worldwide, and alcohol consumption is an important factor in the global burden of disease and disability [1]. Experts continue to warn the public that drinking alcohol brings no health benefits [2,3]. Various countries around the world, and international organizations such as the World Health Organization (WHO), are adopting various positive measures to control alcohol consumption. Yet global alcohol consumption is still on the rise [4]. Moreover, this increase is projected to continue until at least 2030 [5]. As a result, several scholars have warned that the global goal of reducing alcohol damage may prove unsuccessful due to increasing alcohol intake [6]. With this in mind, many scholars have focused on the social factors behind drinking behavior, and believe that studying the social determinants is critical to eliminate alcohol-related harm [7,8,9].

In social psychological research, social discrimination is frequently operationalized as favoring one’s own group over a relevant outgroup. As a result, people in the outgroup receive unfair treatment. In the study of the social determinants of drinking behavior, the existing literature focuses on two areas: social pressure [10,11] and socioeconomic status [12,13]. When facing social discrimination, people often relieve the pressure by consuming alcohol. A study of American college students of African descent showed that the feeling of racial discrimination is likely to lead to negative emotion-related drinking behavior among these students [14]. Outside of racial discrimination, general social discrimination has also been shown to promote college students’ drinking behavior in the United States [15]. For black Americans, both racial and socioeconomic discrimination increase their alcohol intake [16]. According to the stress-coping hypothesis, drinking behavior is an individual’s coping strategy to deal with social discrimination [17]. Therefore, there is a positive correlation between social discrimination and alcohol intake [18]. In the West, in addition to the United States, evidence of social discrimination resulting in drinking can be found in countries such as the United Kingdom [19], the Netherlands [20], and Brazil [21]. Of course, in the West, there was also early evidence that did not support the stress-coping hypothesis [22,23]. However, in the East, there has been little research or empirical evidence, except a few studies in Hong Kong [24,25]. Ancient China was one of the first places in the world where alcohol was distilled. Today, China accounts for one-fifth of the world’s population, and it has a drinking culture of over 5000 years. However, there has been no research on the relationship between social discrimination and drinking in China. This means that the stress-coping hypothesis of drinking behavior lacks important evidence.

Socioeconomic factors have significant effects on alcohol consumption, and this induces alcohol-related diseases [26]. Studies in Chile and Finland have shown that people with low socioeconomic status (SES) have higher rates of alcohol abstinence, but also have higher rates of episodic drinking [27]. In addition, income has a significant effect on the frequency of drinking [28]. However, studies have shown that for 15-year-olds, there is no obvious socioeconomic model to explain drinking behavior [29]. Thus, it can be said that the effect of social inequality on drinking behavior is inconsistent across countries and age groups. Individual differences are also an important factor in drinking behavior. Common personal factors related to drinking include gender, age, ethnicity, education level, and marital status [30,31,32,33]. In terms of geographic area of residence, several studies have pointed out that it has little effect on drinking behavior [34]. This shows that the effect of regional development disparities on drinking behavior is complex, and urban–rural differences exacerbate the complexity [35].

Since the 1980s, alcohol consumption in China has been on the rise. According to the *Global Status Report on Alcohol and Health 2018* published by the WHO, China’s per capita alcohol consumption rose from 4.1 L in 2005 to 7.2 L in 2016. China’s rate of lifelong alcohol abstinence also dropped from 50.9% in 2005 to 42.1% in 2016. However, controversy remains over how socioeconomic factors influence drinking behavior in China. Studies have shown that education, employment, marital status, and regional differences affect alcohol consumption, but the effect of income is not statistically significant [36]. Other studies have shown that income and geographic location have significant effects on drinking behavior, while other socioeconomic factors are insignificant [37]. Studies have also shown that in China, income is positively correlated and education level is negatively correlated with alcohol consumption [38]. However, these studies analyzed the influence of social and economic factors on drinking behavior, but ignored the influence of social pressure factors such as social discrimination due to household registration or gender and unfair treatment by the government. This has led to inconsistencies across the research results. Here, we propose the following hypotheses:

**Hypothesis** **1.** *Perceived social discrimination due to household registration is positively related to adult alcohol consumption.*

**Hypothesis** **2.** *Perceived social discrimination due to gender is positively related to adult alcohol consumption.*

**Hypothesis** **3.** *The experience of conflict with government officials is positively related to adult alcohol consumption.*

**Hypothesis** **4.** *The experience of delays in government interactions is positively related to adult alcohol consumption.*

Alcohol consumption threatens physical and mental health. China has one of the highest levels of alcohol consumption worldwide, as well as the highest number of deaths due to alcohol. Although Chinese government departments have implemented interventions to curb alcohol consumption, drinking behavior has shown no significant improvement. The aim of the present study is to evaluate the effect of perceived social discrimination on drinking behavior, then examine the influence of regional factors, socioeconomic status, and individual differences on alcohol consumption.

### Key Points

1. The alcohol consumption rate among Chinese men is about 13 times that of Chinese women.
2. People with an annual income between 50,000 and 150,000 yuan are more prone to alcoholism than those with income under 50,000 yuan.
3. Groups that have experienced unequal treatment in public services are more prone to alcoholism than those who do not suffer such unequal treatment.

## 2. Methods

### 2.1. Sample

The data used in this study were derived from the China Family Panel Studies (CFPS), a comprehensive longitudinal tracking survey conducted by the China Social Science Research Center (ISSS) at Peking University. The center aims to reflect Chinese society and economy by tracking data at the individual, family, and community level to capture changes in population, education, and health. The CFPS sample covers the populations of 25 provinces/cities/autonomous regions in mainland China, with the exception of Xinjiang, Tibet, Qinghai, Inner Mongolia, Ningxia, and Hainan. Their population accounts for 95% of China’s total population. Therefore, the CFPS sample can be regarded as nationally representative. CFPS mainly conducts face-to-face interviews aided by computer-assisted personal interviewing (CAPI). In situations where personal interviews are not feasible, telephone interviews using computer-assisted telephone interviewing (CATI) or Web interviews using computer-assisted Web interviewing (CAWI) are substituted. There are 4 versions of the CFPS questionnaire: community, family, adult, and child questionnaires. This study used data collected through the adult questionnaire from the 2016 follow-up survey. In that survey, the adult dataset included individuals who were age 16 or over in the previous survey and individuals who were age 16 or over among newly added family members in 2016, for a total of 36,892 adults interviewed. In this study, we obtained a baseline study sample (*n* = 24,032) by matching the 2010 and 2016 data with a personal code. This included all individuals who responded to the 2016 follow-up survey and had been included in the original sample. Subsequently, we removed 1466 individuals with a problem response rate of less than 40% in the 2016 follow-up survey. The processed sample (*n* = 22,566) provided the data for this study.

### 2.2. Measure

#### 2.2.1. Alcoholism and Dependent Variables

“Regularly drink” means more than 3 drinks a week, at least 1 drink each time. We define people who are prone to alcoholism as those who regularly drink, i.e., have a tendency to become regular alcohol users. Respondents were asked “In the past month, did you drink more than 3 times a week?” to get the distribution of the dependent variable. A positive answer is defined as 1 and a negative answer is 0.

#### 2.2.2. Measurement Variables of Perceived Social Discrimination

Growing evidence suggests that chronic exposure to unfair treatment or day-to-day discrimination increases the risk of poor health and alcohol consumption [39,40]. This study used a set of 2-category variables related to public services to measure unfair treatment by the government. This includes whether people have experienced personal injustice due to household registration, unfairness due to gender, conflict with government officials, or delays in interactions with government institutions. For example, respondents were asked: “In the past year, did you receive any unfair treatment due to household registration status?” A positive answer is defined as 1 and a negative answer is 0.

#### 2.2.3. Measurement Variables of Socioeconomic Status

Income is an indicator of socioeconomic status [36,38]. We divided annual income into 3 levels: 0–49,999, 50,000–149,999, and 150,000+ yuan. To account for personal differences, we considered the variables gender, age (16–34, 35–49, 50+), education level (lower, medium, high), employment (employed, unemployed), marital status (married, other marital status), chronic disease within previous 6 months (chronic disease, no chronic disease), and health status (unhealthy, fair, healthy).

#### 2.2.4. Measurement Variables of Regional Variable

Regional variables were divided into western, eastern, and central China (west vs. east vs. central) according to the degree of regional economic development. East included Liaoning, Beijing, Tianjin, Hebei, Shandong, Jiangsu, Shanghai, Zhejiang, Fujian, and Guangdong; central included Shanxi, Jilin, Heilongjiang, Anhui, Jiangxi, Henan, Hubei, and Hunan; and west included Shaanxi, Gansu, Sichuan, Chongqing, Yunnan, Guizhou, and Guangxi. Regional inequality is also reflected in the urban–rural divide. Accordingly, we measured this through individuals’ *hukou* status (non- agricultural vs. agricultural *hukou*) [38]. (*hukou*, also known as household registration, refers to a legal document prepared by the state administrative agency in charge of household registration to record and retain basic information about the household population. It is also the identity certificate of every citizen. *Hukou* is divided into two types: agricultural and non-agricultural. Agricultural *hukou* refers to residents living in rural areas who rely on their own food production. Non-agricultural *hukou* refers to urban residents who rely on the state to distribute rations.)

### 2.3. Statistical Modelling

After multiple imputations of the missing values in the sample (*n* = 22,566), we used chi-square analysis and *t*-test to describe the binary correlation between the influencing factors and tendency toward alcoholism. In this study, we cleared and processed the data using R3.5.2. Next, multivariate logistic regression analysis was conducted to test each hypothesis in the theoretical model using SPSS statistical software (version 25.0, IBM Corp., Armonk, NY, USA), with *p*-value < 0.05 indicating statistical significance. The significance level was set at α = 0.05, and all tests were two-tailed.

### 2.4. Ethical Statement

This research was funded by the Social Science Foundation of China (17BSH122). The authors declare that they have no conflict of interest. Because the data in this research were not collected from human subjects and do not involve human participants or animals, ethical approval was not needed in this research.

#### Ethics Approval and Consent to Participate

The raw data of CFPS were used for this study with permission from the Institute of Social Survey Study, Peking University (ISSS). Informed consent was obtained from all study participants by ISSS.

## 3. Results

The alcohol drinking behavior of respondents is listed in Table 1. In 2016, 15.5% of respondents drank more than three times a week in the previous month (*n* = 3493), while 84.5% drank less than that (*n* = 19,073).

Table 1 also shows a combination of alcoholism-prone and personal characteristics in 2016. The binary cross-tabs show that people with a tendency toward alcohol overconsumption were more likely to be in eastern China, male, and married, compared to those who did not have this tendency. Older individuals were also more likely to abuse alcohol. Additionally, those who thought they were in good health were more likely to abuse alcohol than those who thought they were in average or poor health. Those who had been diagnosed with a chronic disease by a doctor within the past six months were less likely to have a tendency toward alcohol overconsumption than those who had not.

Those who had experienced unfairness due to household registration, conflicts with government officials, or delays in interactions with government were more likely to drink alcohol than those who had not experienced these unfair treatments. On the contrary, those who had experienced gender discrimination had lower rates of alcohol consumption than those who had not. Furthermore, there were no statistically significant differences in alcohol consumption across different types of household registration.

Table 2 presents the results of multivariate logistic regression analysis. Cox and Snell R square is 0.117 and *ρ*^2^ (Nagelberke) is 0.252. The results of this analysis show that in 2016, people in eastern China were more alcohol-prone than those in western China (odds ratio (OR) = 1.812; 95% confidence interval (CI): 1.614–2.035). Furthermore, people in central China were more alcohol-prone than those in western China (OR = 1.683; 95% CI: 1.482–1.911). Men were more alcohol-prone than women (OR = 13.006; 95% CI: 11.438–14.438). People aged 50 and above were more likely to abuse alcohol than those aged 16–34 (OR = 2.073; 95% CI: 1.549–2.775). Furthermore, people aged 35–49 were more likely to abuse alcohol than those aged 16–34 (OR = 1.677; 95% CI: 1.443–1.948). Those with a higher education were less alcohol-prone than those with less education (OR = 0.461; 95% CI: 0.329–0.646). Further, those with medium-level education were less alcohol-prone than those with less education (OR = 0.758; 95% CI: 0.666–0.862). Those with total income between 50,000 and 150,000 yuan were more alcohol-prone than those with total annual income below 50,000 yuan (OR = 1.338; 95% CI: 1.051–1.703), while those with total income more than 150,000 yuan were no more alcohol-prone than those with total annual income below 50,000 yuan (OR = 0.634; 95% CI: 0.334–1.201). Married people were more alcohol-prone than those with other marital status (OR = 1.174; 95% CI: 1.034–1.332). Those who had been diagnosed with a chronic disease within the last six months were less likely to abuse alcohol than those who had not (OR = 0.702; 95% CI: 0.625–0.790). The better people’s health status was, the more likely they were to have abused alcohol (OR = 1.219; 95% CI: 1.149–1.928).

Those who experienced unfair treatment due to household registration had a negative statistically significant tendency toward alcohol overconsumption (OR = 0.817; 95% CI: 0.682–0.980). Thus, Hypothesis 1 was not supported. Those who had experienced unfair treatment due to gender were more prone to alcohol overconsumption than those who had not (OR = 1.486; 95% CI: 1.145–1.928). Thus, Hypothesis 2 was supported. Conflict with government officials had no significant effect on tendency toward alcohol overconsumption when compared to no conflict (OR = 1.147; 95% CI: 0.943–1.394). Thus, Hypothesis 3 was not supported. People who had experienced delays in interacting with the government showed a tendency for alcohol overconsumption compared to those without such experience (OR = 1.119; 95% CI: 1.068–1.345). Thus, Hypothesis 4 was supported.

There was not a higher alcohol-prone tendency among those who were employed than those who were unemployed (OR = 1.258; 95% CI: 0.613–2.582). The type of household registration had no significant correlation with alcohol consumption (OR = 0.939; 95% CI: 0.840–1.051). For a further robustness test, we used the panel data of this sample in 2010 and ran multivariate logistic regression analysis again. The results supported the previous conclusions, except for two independent variables. In 2010, employment status and type of household registration had a significant correlation with alcohol consumption. It shows that the influence of these two independent variables on adult drinking behavior is unstable.

## 4. Discussion

According to the stress-coping hypothesis of drinking behavior, when individuals feel social discrimination or unfair treatment, they will feel social pressure and turn to drinking as a coping strategy [10,11]. This study’s results show that perceived gender discrimination and delays in government interactions as a result of unfair treatment have a positive and significant effect on an individual’s drinking; that is, these types of perceived social discrimination result in social pressure, and individuals will often choose to drink to relieve it. Therefore, drinking is a strategy to deal with perceived social pressure. This also shows that this kind of perceived social discrimination can lead to negative emotions, and drinking is a common coping strategy to mitigate negative emotions [14]. When an individual is exposed to regular unfair treatment and discrimination over a long period of time, it not only promotes an increase in alcohol consumption, but also has a negative effect on the individual’s health [39]. Although such unfair treatment and perceived social discrimination are often invisible and unlikely to cause widespread public concern, the negative effects on individual drinking behaviors and health are often long-term and long-lasting. Therefore, such perceived social discrimination acts as an obstacle to meeting the public health goal of reducing alcohol harm.

Of course, whether the stress-coping hypothesis holds true is also related to the type of stress. The results of this study show that pressure from conflict with government employees has no significant relationship with drinking behavior. In other words, this pressure does not affect individual drinking behavior, and individuals do not use alcohol to relieve or cope with this pressure. In addition, the findings also show that perceived discrimination due to *hukou* household registration has an inhibitory effect on individual drinking behavior; that is, there is a negative correlation between drinking and perceived discrimination due to household registration. This negative correlation between perceived social pressure and drinking behavior also appeared in early Western literature [22,23]. This shows that perceived social pressure does not necessarily increase drinking behavior, and may even have an inhibitory effect. In China, the negative effect of household registration discrimination on drinking behavior is a function of the long-standing urban–rural differences in Chinese society—the so-called urban–rural dual structure system [38]. This system is a serious obstacle to China’s economic and social development. The preeminent manifestation of this structure is the *hukou* barrier between urban and rural areas, i.e., different resource allocation systems and other issues. As this urban–rural dual structure system has existed for decades and is entrenched, discrimination based on household registration remains prevalent [38]. This study shows that the stress-coping hypothesis test for drinking behavior must be analyzed within the context of a specific social structures and systems. The effect of perceived social pressure on drinking behavior will also be mediated by social and economic structures and systems.

According to relative deprivation theory, objective discrimination by the government causes people to form grievances and drink alcohol or engage in political activities against the government to cope with social stress [41]. In our study, the experience of conflict with government officials was used to measure objective discrimination by the government. We found that more experience of conflict with government officials did not predict more social pressure and alcohol drinking. An important reason for this difference is the difference between Eastern and Western cultures, which gives Chinese and Western individuals different perceptions of and coping strategies for social pressure [42,43]. Therefore, when analyzing the impact of perceived social discrimination on individual drinking behavior, it is necessary to consider the differences between Chinese and Western cultures. In the West, drinking is often a way to release the pressure brought by work and life. In China, drinking is often used as the main means of social entertainment and gatherings of friends and family.

In terms of regional inequality, the tendency to use alcohol regularly in eastern China is higher than in central China, and the tendency in central China is higher than in western China. The division into eastern, central, and western regions is based on the geographic location of each province and its economic development. On the whole, eastern China is more economically developed than central China, and central China is more economically developed than western China. In other words, the tendency to use alcohol regularly is higher in areas with strong economic development than in underdeveloped areas. In addition, there is no significant difference between alcohol consumption patterns in agricultural and non-agricultural households, in terms of household registration types. China’s household registration is divided into agricultural and non-agricultural *hukou*. Broadly speaking, these represent rural and urban residents. The statistically insignificant results show that there is no urban–rural difference in alcohol consumption.

Among the individual difference variables, the results show that in terms of gender, the tendency of men to use alcohol regularly was about 13 times that of women. In the research on drinking in other countries, men consistently outnumber women in terms of drinking frequency, amount of alcohol consumed, incidence of heavy drinking, and the consequences of heavy drinking [44]. The differences stem from men’s and women’s different needs and motivations for drinking. In many cultures, alcohol is a powerful symbol of gender roles and identities, and social structural norms maintain this gender difference [45].

In terms of age, the tendency of people older than 50 years to use alcohol regularly was higher than that of people 35–49 years old, and the tendency of those 35–49 years old is higher than that of those 16–34 years old. This demonstrates a trend of increasing alcohol consumption with age. The influence of the employment variable on the tendency to use alcohol regularly was insignificant. In terms of education level, those with low education levels were more likely to use alcohol regularly than those with higher education levels. In 2016, the rate for those with a high level of education was 46.1% of the rate of those with a low level; the rate among people with a medium level was 75.8% of the rate of those with a low level. These findings are similar to Cawley and Ruhm ’s conclusion that there is an inverse relationship between alcoholism and education [46]. However, these results are contrary to those of Marques-Vidal and Dias, who found that light drinking was more common among people with higher education in Portugal than among those without higher education [47]. Jonas et al. also showed that in Australia, highly educated women were more likely to drink than women with less education [48]. These differences show that the effects of education on alcohol consumption vary across countries. In China, these results underscore the idea that policymakers should increase the proportion of the population that receives secondary or higher education, and expand educational opportunities for those with little education. This could reduce the proportion of people who abuse alcohol. It could also potentially reduce social problems such as violence, drug abuse, and crime, in which alcohol dependence can be a factor.

In terms of marital status, numerous studies have shown that married people are less likely to drink alcohol [49,50]. There are two reasons for this. First, marriage has obvious health benefits. Married people have more health-promoting behaviors and avoid lifestyles that may increase their risk of disease. This includes drinking, and especially alcoholism [51]. Second, heavy drinkers have a slim chance of getting married due to screening [50]. The present study reaches the opposite conclusion. The data show that the tendency to use alcohol regularly among people who are married tends to be higher compared to others. This may be due to increased stress after marriage. Marriage entails additional social roles. Not only does this change in the relationship structure bring pressure, but the social complications it causes can also raise difficulties. That is, though marriage can produce an intimate relationship, it also breeds conflict. Factors such as children or an unequal division of labor in the family may intensify marital conflict, thus reducing happiness. The consumption of alcohol can be an outlet to relieve tension or stress. In response, community social workers can intervene to advise families experiencing conflict, and ease disagreement and friction within the family. This type of intervention could also reduce the rate of alcoholism among this group.

In terms of health status, the tendency to use alcohol regularly among people who had been diagnosed by a doctor with a chronic disease within the past six months was lower compared to those who had not. In 2016, the odds ratio was 0.702. People with chronic diseases need frequent interaction with doctors, and doctors’ advice can influence their behavior. It stands to reason that if doctors often remind chronic patients that they should not drink alcohol, the patients will avoid exposure to alcohol in their daily lives so that they can recover faster. The findings of Kenkel and Terza [52] also demonstrate that doctors’ advice is a more effective and less costly intervention for patients with alcohol-related problems. Therefore, service personnel at medical institutions can remind their patients more often of the hazards of long-term alcohol overconsumption. This can also have a positive effect on reducing the proportion of alcoholics.

In addition, people with a self-rated health status of healthy had a higher tendency to use alcohol regularly than those with poor health status. However, the subjective evaluation of one’s own health status might not be an accurate reflection of health. For example, if someone feels that their body is healthy, they may drink alcohol at will. This will increase the risk of illness. Therefore, social services and government departments should promote physical health and guide the public’s self-care awareness.

Regarding income, we found that people with an annual income between 50,000 and 150,000 yuan had a higher tendency to use alcohol regularly than people with income below 50,000 yuan. There was no significant difference between people who earned more than 150,000 yuan and those who earned less than 50,000 yuan in terms of alcohol consumption.

This study has its limitations. First, drinking behavior is based on self-reported data. Social expectations sometimes create a gap between self-reported data and actual drinking frequency. Second, there may be an interaction between social discrimination and other socioeconomic variables. Third, despite the drinking frequency data in the dependent variable, there is a lack of data measuring alcohol intake. This makes it difficult to assess drinking severity and distinguish between serious and general drinking behavior. Despite these limitations, this study provides new evidence for the relationship between social pressure and drinking behavior. Furthermore, this study sheds new light on drinking behavior under China’s social and economic system. It can be a useful reference in efforts to curb drinking-related adverse health outcomes.

In the 2016 CFPS adult questionnaire, there was only one question about drinking behavior: “Did you drink alcohol at least three times a week in the past month?”. The answer was yes or no, so it is impossible to further distinguish the specific types of adult drinking. The patterns of consumption in China can be classified into five subtypes: abstainers, ex-weekly drinkers, reduced-intake drinkers, occasional drinkers, and current weekly drinkers [53]. We suggest that the adult drinking questions in the CFPS survey should be designed with more detail based on this classification, so that a more in-depth and detailed study can be carried out. Moreover, if the type of alcohol and frequency of drinking were included in the CFPS survey, we could estimate weekly alcohol intake and accurately assess the impact of perceived social discrimination on adult alcohol intake. These are the directions for future research.

## 5. Conclusions

Based on data from a nationally representative survey in China, this study’s results found that everyday unfair treatment and perceived discrimination such as being discriminated against because of gender and facing delays in government interactions are associated with a higher tendency to use alcohol regularly. However, being discriminated against because of one’s *hukou* was associated with a lower tendency of using alcohol regularly and experiencing conflict with government officials was not associated with alcohol consumption. From the perspective of socioeconomic status, income has a significant effect on adult drinking. Unlike drug treatment for patients with alcohol dependency, this study proposes management methods such as policy adjustments and community interventions for people with alcoholic tendency. This can reduce the cost of social governance.

## Figures and Tables

**Table 1:** Distribution and chi-square of general characteristics.

Classification		Number of Participants (*n* = 22,566)	Drink or Not		χ^2^ (*p*)
		*n* (%)	Drink *n* (%)	Do Not Drink *n* (%)	
District	Eastern region	9551 (42.3)	1599 (45.8)	7953 (41.7)	47.35 *** (0.000)
	Central region	6637 (29.4)	1073 (30.7)	5564 (29.3)	
	Western region	6348 (28.1)	818 (23.4)	5530 (29.0)	
Gender	Male	10,927 (48.4)	3148 (90.1)	7779 (40.8)	2877.49 *** (0.000)
	Female	11,639 (51.6)	345 (9.9)	11,294 (59.2)	
Hukou	Agricultural	16,189 (71.8)	2538 (72.7)	13,651 (71.6)	2.24 (0.524)
	Non-agricultural	6372 (28.2)	955 (27.3)	5417 (28.4)	
Age	16–35	3665 (16.2)	368 (10.5)	3297 (17.3)	102.77 *** (0.000)
	36–50	6480 (28.7)	1025 (29.3)	5455 (28.6)	
	>50	12,421 (55.0)	2100 (60.1)	10,321 (54.1)	
Highest level of Education	Primary school and below	11,547 (52.7)	1706 (50.2)	9841 (53.1)	67.64 *** (0.000)
	High school	8474 (38.6)	1491 (43.9)	6983 (37.7)	
	3-year college and above	1910 (8.7)	203 (6.0)	1707 (9.2)	
Income	0–50,000 yuan	2759 (79.3)	557 (78.2)	2202 (79.5)	1.14 (0.565)
	50–150,000 yuan	691 (19.9)	150 (21.2)	541 (19.5)	
	>150,000 yuan	31 (0.9)	5 (0.7)	26 (0.9)	
Employed or not	Yes	383 (8.7)	32 (7.4)	351 (8.9)	1.05 (0.306)
	No	3995 (91.3)	399 (92.6)	3596 (91.1)	
Marital status	Married	19,158 (84.9)	3094 (88.6)	16,064 (84.3)	43.46 *** (0.000)
	Other	3407 (15.1)	399 (11.4)	3008 (15,8)	
Chronic disease	Yes	4364 (19.3)	482 (13.8)	3882 (20.4)	81.38 *** (0.000)
	No	18,199 (80.7)	3011 (86.2)	15,188 (79.6)	
Health	Unhealthy	8543 (37.9)	1091 (31.2)	7452 (39.1)	86.44 *** (0.000)
	Fair	7899 (35.0)	1288 (36.9)	6611 (34.7)	
	Healthy	6124 (27.1)	1114 (31.9)	5010 (26.3)	
Hukou discrimination	Yes	1379 (6.1)	221 (6.3)	1158 (6.1)	0.33 (0.566)
	No	21,178 (93.9)	3273 (93.7)	17,906 (93.9)	
Gender discrimination	Yes	703 (3.1)	101 (2.9)	602 (3.2)	0.69 (0.405)
	No	21,855 (96.9)	3392 (97.1)	18,463 (96.8)	
Conflict with officials	Yes	920 (4.1))	190 (5.4)	730 (3.8)	19.58 *** (0.000)
	No	21639 (95.9	3303 (95.9)	18336 (96.2)	
Government delay	Yes	3252 (14.4)	638 (18.3)	2614 (13.7)	49.61 *** (0.000)
	No	19,305 (85.6)	2855 (81.7)	16,450 (86.3)	
Drink or not	Yes	3493 (15.5)	-	-	-
	No	19,073 (84.5)	-	-	

Note: *** significant at 1%.

**Table 2:** Multivariable logistic regression analysis.

Characteristics	Drink_Y_16		
	Odds Ratio	95% CI	*p*-Value
District			
Eastern vs. western	1.812	1.614–2.035	0.000
Central vs. western	1.683	1.482–1.911	0.000
Gender: male vs. female	13.006	11.438–14.438	0.000
Hukou: non-agricultural vs. agricultural	0.939	0.840–1.051	0.271
Age group			
35–49 vs. 16–34 years	1.677	1.443–1.948	0.000
50+ vs. 16–34 years	2.073	1.549–2.775	0.000
Education			
High vs. low	0.461	0.329–0.646	0.000
Medium vs. low	0.758	0.666–0.862	0.000
Personal income			
50,000–149,999 vs. 0–49,999 yuan	1.338	1.051–1.703	0.018
150,000+ vs. 0–49,999 yuan	0.634	0.334–1.201	0.159
Employed: yes vs. no	1.258	0.613–2.582	0.434
Marital status: married vs. other	1.174	1.034–1.332	0.013
Chronic disease: yes vs. no	0.702	0.625–0.790	0.000
Health (unhealthy/fair/healthy)	1.219	1.149–1.928	0.000
Hukou discrimination: yes vs. no	0.817	0.682–0.980	0.029
Gender discrimination: yes vs. no	1.486	1.145–1.928	0.003
Conflict with officials: yes vs. no	1.147	0.943–1.394	0.169
Government delay: yes vs. no	1.199	1.068–1.345	0.002
Constant	0.006	0.004–0.010	0.000
Chi-square (sig.)	483.728 (0.000)		
-2Log likelihood	15887.96		
Cox and Snell R square	0.117		
*ρ*^2^ (Nagelberke)	0.252		
